# Relationship between Personality Disorder Functioning Styles and the Emotional States in Bipolar I and II Disorders

**DOI:** 10.1371/journal.pone.0117353

**Published:** 2015-01-27

**Authors:** Jiashu Yao, You Xu, Yanhua Qin, Jing Liu, Yuedi Shen, Wei Wang, Wei Chen

**Affiliations:** 1 Department of Psychiatry, Sir Run Run Shaw Hospital, Zhejiang University College of Medicine, Hangzhou, China; 2 Department of Clinical Psychology and Psychiatry/ School of Public Health, Zhejiang University College of Medicine, Hangzhou, China; 3 Department of Diagnostics, Hangzhou Normal University School of Medicine, Hangzhou, China; Catholic University of Sacred Heart of Rome, ITALY

## Abstract

**Background:**

Bipolar disorder types I (BD I) and II (BD II) behave differently in clinical manifestations, normal personality traits, responses to pharmacotherapies, biochemical backgrounds and neuroimaging activations. How the varied emotional states of BD I and II are related to the comorbid personality disorders remains to be settled.

**Methods:**

We therefore administered the Plutchick – van Praag Depression Inventory (PVP), the Mood Disorder Questionnaire (MDQ), the Hypomanic Checklist-32 (HCL-32), and the Parker Personality Measure (PERM) in 37 patients with BD I, 34 BD II, and in 76 healthy volunteers.

**Results:**

Compared to the healthy volunteers, patients with BD I and II scored higher on some PERM styles, PVP, MDQ and HCL-32 scales. In BD I, the PERM Borderline style predicted the PVP scale; and Antisocial predicted HCL-32. In BD II, Borderline, Dependant, Paranoid (-) and Schizoid (-) predicted PVP; Borderline predicted MDQ; Passive-Aggressive and Schizoid (-) predicted HCL-32. In controls, Borderline and Narcissistic (-) predicted PVP; Borderline and Dependant (-) predicted MDQ.

**Conclusion:**

Besides confirming the different predictability of the 11 functioning styles of personality disorder to BD I and II, we found that the prediction was more common in BD II, which might underlie its higher risk of suicide and poorer treatment outcome.

## Introduction

Bipolar I (BD I) and II (BD II) disorders are considered in one disease spectrum, but BD II patients are characterized by recurrent depressive episodes interspersed with hypomania instead of mania which BD I patients often have [1]. Although there might be similarities in managing patients with BD I and II disorders, these patients responded differently to either pharmacologic or other treatments. Generally, lithium and antidepressants have superior benefits in BD II than those in BD I [2, 3]. Specifically, for the rapid cycling state, lamotrigine is effective in BD II but not in BD I [4]; for the memory problems, donepezil is effective in BD II but not in BD I [5]. Moreover, for patients with resistance to pharmacotherapies, the electroconvulsant therapy is more effective in BD II than that in BD I [6]. Scholars are wondering what mechanisms are behind the different management effects in these two types of bipolar disorder [4].

Biological studies for example, have shown some promises in this regard. Although several studies have suggested a unique genetic distinction between BD I and II disorders [7–11], the serotonin transporter binding sites in the midbrain of patients with BD I in the euthymic state are significantly lower than those in BD II [12]. Neuroimaging techniques on the other hand, have detected widespread gray matter reductions in the bilateral frontal, temporal, parietal and parahippocampal areas in BD I patients rather than in BD II [13]. Further in clinics, BD II patients have a more chronic course, mainly with major and minor depressive episodes and shorter interepisodes, while BD I patients have one or more manic or mixed episodes [14–16]. Seasonality and the rapid-cycling course are more pronounced in BD II [14]. Patients with BD II have been hospitalized and presented psychotic symptoms less frequently [16], and exhibited a higher trend of lifetime co-occurrence of phobia [14, 15]. The risk of suicide is higher [14, 17], and the treatment outcome is poorer [18] in BD II than those in BD I.

The above-mentioned differences between BD I and II disorders might be related to the personality variance, although the common denominator for these differences is unclear, and relationships between biological, clinical and personality features are mutual. One study focusing the normal personality traits has found that neuroticism was higher and extraversion was lower in BD II than those in BD I [19]. However, the normal personality trait might only contribute to an increased likelihood of depression, instead of mania, in the Korean BD I patients [20]. The personality disorders might be more closely related to the clinical symptoms of bipolar disorder, since about 40–50% of bipolar patients have fulfilled the criteria for a personality disorder [21]. Bipolar disorder patients who were comorbid with personality disorders had severer mood symptoms, lower level of social function, and more suicidal ideations and behaviors than those who were not [22–24]. Patients with these comorbidities also spent more hospitalized days in a given year [25], and were less likely to achieve symptomatic recovery [26]. The Clusters B (dramatic, emotionally erratic) and C (fearful, avoidant), rather than Cluster A (odd, eccentric), personality disorders were more often comorbid with bipolar disorders [27]. Furthermore, mania score was only positively related to Cluster B personality disorder scores, and depression score were only positively correlated with Cluster C personality disorder scores. Due to common clinical features such as affective instability and impulsivity, borderline personality disorder could be misdiagnosed as bipolar disorder [28–30]. There are also similarities of the causes, courses, and treatments between the two disorders [31, 32]. Moreover, BD II shows a stronger association with borderline personality disorder than with BD I [33, 34]. In addition, scholars have noticed a hypomanic personality, which was characterized by persistently high levels of energy, sociability, confidence, activity and achievement orientation [35], and a cognitive style, which was manifested by the entitlement/ grandiosity, insufficient self-control, and the absence of emotional inhibition [36, 37], those were linked to the development of bipolar disorder. However, these studies have not assessed the relationship between different emotional states of BD I, BD II and personality disorders (including the borderline type) in depth.

Therefore in the current study, we aimed to explore how personality disorder functioning styles contribute to the emotional symptoms (i.e., mania, hypomania and depression) of BD I and II disorders. Since BD I has higher level of extraversion [19], which is correlated with the gregariousness personality disorders [38], these patients might present more comorbidities of the histrionic and narcissistic personality disorders. BD II has higher level of neuroticism [19] which is related to the emotional distress personality disorders [38]; these patients might present more comorbidities of the borderline, avoidant, dependent, paranoid, and schizotypal personality disorders. Moreover, scholars believe that BD I and II disorders represent the manic and depressive extremes respectively [15]. We might further hypothesize that the Cluster B (antisocial, borderline, histrionic and narcissistic) personality disorders are positively related to mania and hypomania in BD I, and the Cluster C (avoidant and dependent) personality disorders are positively correlated with depression in BD II.

Inspired by previous investigations [39–42], we have administered the Mood Disorder Questionnaire [43] and the Hypomanic Checklist-32 [44] for the measures of mania and hypomania, the Plutchik – van Praag Depression Inventory [45] for depression, and the Parker Personality Measure [46] for personality disorder functioning styles in our participants.

## Methods

### Participants

Thirty-seven patients with bipolar disorder type I (BD I; 20 women and 17 men; mean age, 23.22 years with 5.44 S.D.; age range, 17–41 years) and thirty-four with bipolar disorder type II (BD II; 26 women, 8 men; mean age, 24.88 ± 4.58, range, 17–33) were enrolled in the current study. Among them, 11 BD I and 26 BD II patients were comorbid with different types of personality disorders (Table 1). Some patients were comorbid with other psychiatric disorders, such as anxiety, obsessive-compulsive disorder, paranoid state anxiety, etc., but not schizophrenia or schizoaffective disorders. All patients were firstly diagnosed according to the DSM-IV-TR [47] and later confirmed according to the DSM-5 [1] by two experienced psychiatrists (WC and WW). In addition, they suffered from no organic brain lesions according to the recent magnetic resonance imaging or computed tomography scans, and had to be free from antipsychotic drugs or alcohol for at least 72 hours prior to testing. Seventy-six healthy participants (49 women and 27 men; mean age, 22.74 ± 3.44, ranged, 20–38) were also enrolled, who had no history of psychiatric or neurological abnormalities, and also were free from alcohol or drug use at least 72 hours prior to participating in the study. No significant difference was found among the three groups regarding either gender (χ2 = 3.60, p >.05) or age (F [2, 144] = 2.97, p > 0.05, MSE = 2749.61) distributions. The study protocol was approved by the Medical Ethics Committee of School of Public health, Zhejiang University and all participants had given their written informed consents. For participants of 17 years old, we have obtained the written informed consent by their next of kin, through a surrogate consent procedure, regarding participating in our study.

**Table 1:** Numbers (in brackets) of BD I (n = 37) or II (n = 34) patients comorbid with personality disorder.

	**Cluster A**	**Cluster B**	**Cluster C**	**Multiple**
BD I	schizoid (2)	borderline (1)	dependent (1)	mixed (4)
		histrionic (2)	obsessive-compulsive (1)	
BD II	schizoid (3)	histrionic (8)	dependent (2)	mixed (4)
	schizotypal (2)	antisocial (3)	obsessive-compulsive (1)	
	paranoid (1)	borderline (1)	avoidant (1)	

Note: mixed, more than two types of personality disorder each from cluster A, B or C.

### Measures

Participants were asked to complete the following four questionnaires in a quiet room.

A. The Mood Disorder Questionnaire (MDQ) [43], which consists of three parts, including 13 forced-choice (yes or no) questions to assess the presence of symptoms and behaviors related to mania or hypomania, one question to determine whether two or more symptoms have been experienced at the same time, and one question to determine the extent to which symptoms have caused functional impairment on a scale ranging from “no problems” to “serious problems”. According to a recent study [39], its internal reliability was.79.

B. The Hypomania Checklist-32 (HCL-32) [44], which is a self-assessment instrument comprising 32 items for detecting hypomanic symptoms. Individuals were instructed to answer the forced-choice (yes or no) questions about emotions, thoughts, or behaviors, and to answer questions regarding the duration, the impact of family, social and work life, or people’s reactions. According to a recent study [40], its internal reliability was.88 in a Chinese sample.

C. The Plutchik—van Praag Depression Inventory [45], which consists of 34 items. Each item has three scale points (0, 1, 2) corresponding with increasing depressive tendencies. Subjects have “possible depression” if they score between 20 and 25, or “depression” if they score above 25. According to a recent study [41], the internal reliability of the inventory was.94 in a Chinese sample.

D. The Parker Personality Measure (PERM) [46], which measures 11 functioning styles of paranoid, schizoid, schizotypal, antisocial, borderline, histrionic, narcissistic, avoidant, dependent, obsessive-compulsive, and passive-aggressive personality disorders. Each PERM item has a 5-point Likert scale (1 – very unlike me, 2 – moderately unlike me, 3 – somewhat unlike and like me, 4 – moderately like me and 5 – very like me). Its Chinese version has proven to be reliable in China [42].

### 2.3. Statistics

Two-way ANOVA was applied to the mean PERM style scores (all 11 scales were included), and one-way ANOVA to the mean MDQ, HCL-32 and PVP scale scores in the three groups of participants. Whenever a significant main effect was found, post-hoc analysis by the Bonferroni test (P<.05) was employed to evaluate between-group differences. Inspired by a study [48], we applied the multiple linear regression analysis (backward method) to search for the relationships between the PERM styles, MDQ, HCL-32 and PVP scales, taking PERM styles as potential predictors for the rest scales. A p value less than.05 was considered as significant.

## Results

The mean PVP scores were significantly different among the three groups (F [2, 142] = 33.62, p = 0.00, MSE = 3714.80; the effect survived after controlling for MDQ), with that in BD II higher than those in BD I (p <.05, 95% confidence interval (CI): 9.42 ~ 21.71) and the controls (p <.05, 95% CI: 12.49 ~ 23.22) (Table 2). The mean MDQ scores were significantly different among the three groups (F [2, 140] = 15.46, p = 0.00, MSE = 294.62; the effect survived after controlling for PVP), with that in BD I higher than those in BD II (p <.05, 95% CI: 2.88 ~ 6.39) and the controls (p <.05, 95% CI: 3.20 ~ 6.07). The mean HCL-32 scores were also significantly different among the three groups (F [2, 140] = 42.09, p = 0.00, MSE = 612.84), with those in BD I (p <.05, 95% CI: -8.17 ~ -8.46) and BD II (p <.05, 95% CI: -7.20 ~ -3.21) higher than that in the controls.

**Table 2:** Scale scores (Mean ± S.D.) of the Plutchik-van Praag Depression Inventory (PVP), the Hypomanic Checklist-32 (HCL-32), the Mood Disorder Questionnaire (MDQ) and the Parker Personality Measure (PERM) in the healthy volunteers (Controls, n = 76) and patients with bipolar I (BD I, n = 37) and II (BD II, n = 34) disorders.

	**Controls**	**BD I**	**BD II**
**PVP**	10.33 ± 9.68	12.62 ± 8.34	28.19 ± 14.10a, b
**HCL-32**	16.20 ± 4.41	22.51 ± 2.63a	21.40 ± 3.38a
**MDQ**	4.50 ± 3.49	9.14 ± 1.60a	4.50 ± 2.70b
**PERM**			
Paranoid	23.28 ± 6.69	27.70 ± 7.52a	29.24 ± 6.77a
Schizoid	19.93 ± 4.13	20.62 ± 4.87	21.12 ± 4.70
Schizotypal	10.51 ± 3.13	12.81 ± 4.36a	12.91 ± 4.41a
Antisocial	21.14 ± 5.60	24.54 ± 5.41a	25.94 ± 7.45a
Borderline	21.87 ± 6.22	27.49 ± 7.95a	32.03 ± 8.05a, b
Histrionic	14.36 ± 3.68	16.38 ± 4.34	18.18 ± 3.89a
Narcissistic	18.34 ± 5.22	21.49 ± 5.22a	22.76 ± 5.25a
Avoidant	26.47 ± 7.02	29.65 ± 7.71	32.18 ± 8.45a
Dependent	22.57 ± 6.60	26.57 ± 6.53a	30.29 ± 8.37a
Obsessive-Compulsive	18.11 ± 4.17	19.46 ± 4.45	19.68 ± 5.30
Passive-Aggressive	22.17 ± 5.53	24.03 ± 5.43	25.18 ± 6.77a

Note: a, p <.05 vs. Controls; b, p <.05 vs. BD I.

The mean PERM scale scores were significantly different among the three groups (group effect, F [2, 144] = 19.35, p = 0.00, MSE = 3084.21; scale effect, F [10, 1440] = 165.17, p = 0.00, MSE = 5179.93; group X scale interaction effect, F [20, 1440] = 4.33, p = 0.00, MSE = 135.89) (also see Table 2). Post-hoc analyses showed that groups of BD I and BD II scored significantly higher than the controls did on Paranoid, Schizotypal, Antisocial, Narcissistic, and Dependant scales. BD II scored significantly higher than the controls did on Histrionic, Avoidant, and Passive-Aggressive scales. BD II scored significantly higher on Borderline scale than BD I did, and the latter scored significantly higher than the controls did. The post-hoc between group effects of one PERM scale survived after controlling the rest scales.

When considering the prediction of PVP, MDQ and HCL-32 scales by the PERM styles, results showed that the accounted variance (adjusted R^2^ values) ranged from.02 to.36 in the controls, from.07 to.32 in BD I, and from.24 to.46 in BD II (Table 3). In controls, the Borderline and Narcissistic (-) styles predicted the PVP scale; the Borderline and Dependant (-) styles predicted the MDQ scale. In BD I, the Borderline style predicted the PVP scale; and the Antisocial style predicted the HCL-32 scale. In BD II, the Borderline, Dependant, Paranoid (-) and Schizoid (-) styles predicted the PVP scale; the Borderline style predicted the MDQ scale; Passive-Aggressive and the Schizoid (-) styles predicted the HCL-32 scale (Table 3).

**Table 3:** Backward multiple regression (p to remove was set at.10) of predicting the Plutchik-van Praag Depression Inventory (PVP), the Hypomanic Checklist-32 (HCL-32), and the Mood Disorder Questionnaire (MDQ) by the Parker Personality Measure (PERM) in the healthy volunteers (Controls, n = 76) and patients with bipolar I (BD I, n = 37) and II (BD II, n = 34) disorders.

		**Controls**		**BD I**		**BD II**
	**a-R^2^**	**beta (B, SE), predictor**	**a-R^2^**	**beta (B, SE), predictor**	**a-R^2^**	**beta (B, SE), predictor**
**PVP**	.28	**.46 (.56, .14) Borderline**	.32	**.58 (.61, .15) Borderline**	.46	**.58 (1.02, .31) Borderline**
		.28 (.58, .30) Histrionic				**.41 (.67, .25) Dependent**
		**-.41 (-.58, .18) Narcissistic**				**-.33 (-.66, .32) Paranoid**
						**-.37 (-1.10, .43) Schizoid**
**MDQ**	.11	**.48 (.25, .08) Borderline**	.07	.31 (.12, .06) Histrionic	.24	**.52 (.19, .06) Borderline**
		**-.33 (-.16, .07) Dependent**				
**HCL-32**			.15	.32 (.18, .10) Schizoid	.43	**.32 (.19, .08) Passive-Aggressive**
				**.42 (.21, .08) Antisocial**		**-.65 (-.44, .10) Schizoid**
				-.34 (-.12 .07) Avoidant		

Note: (1) a-R^2^, adjusted R^2^. (2) Tolerance levels: Controls (PVP, .42~.81; MDQ, .58~.88; HCL-32, .84~1), BP I (PVP, .59~.90; MDQ, .67~1; HCL-32, .41~.82), BP II (PVP, .27~.91; MDQ, .68~1; HCL-32, .47~.95). (3) Significant predictors (P<0.05) are bolded for clarity.

## Discussion

Regarding PERM scales, both BD I and II groups scored significantly higher on some of them than the healthy controls did. The BD II group scored also higher than BD I on the Borderline scale. These findings were in line with previous studies [21, 33, 34]. The significant higher Borderline scale came out against the view that twice as many with a diagnosis of BD II as compared to that of BD I comorbid with personality disorder, but unsurprisingly contributed to several correlations with the emotional symptoms in our participants. The BD II group scored significantly higher on PVP than BD I and the healthy controls did. The BD I scored significantly higher on MDQ than both BD II and the controls did. Both BD I and II groups scored significantly higher on HCL-32 than the healthy controls did. These outcomes support that BD I and II represent the manic and depressive extremes in the bipolar spectrum respectively [15]. It is the first time that we applied all the 11 personality disorder functioning styles to predict the emotional states of bipolar disorder. Although the predictions did not fully support our hypothesis, they did present different relationship patterns in the two types of bipolar disorder. Moreover, BD I scored higher on MDQ than BD II did, but scored similarly on PERM scales to BD II did except on Borderline, which might imply that different factors contribute to the unstable moods across the two types of bipolar disorder.

The adjusted R^2^ of predicting PVP by PERM styles were generally higher than those of predicting HCL-32 and MDQ scales, especially in the healthy controls and in BD I groups. The results were in accordance with previous research that the affective disorders with higher personality disorder frequencies, and with higher depression but not mania [21]. The PERM style predictions for HCL-32 were stronger than those for MDQ in patient groups, which was in accordance with the study showing the bipolar disorder patients reported a reduction in maladaptive personality traits when they recovered from the hypomanic episode [49].

In BD I, the Borderline style was the only predictor for PVP scale. Its contribution might be related to the fact that the borderline personality disorder patients are often comorbid with depression [50, 51]. Moreover, the Antisocial style predicted the HCL-32 scale, which was consistent with the phenomena that bipolar disorder patients had less agreeableness trait (the opposite end of an antisocial domain) and lower treatment-compliance in clinics [52]. Another study also has shown that the Antisocial personality disorder was more strongly related to mania than other axis I diseases [53].

In BD II, the Borderline style was correlated with the PVP scale as it was in BD I (see the discussion above). Besides, the Dependent, Schizoid (-) and Paranoid (-) styles contributed to the prediction of PVP. Indeed, patients with dependent personality disorder are prone to depression [54], and patients with schizoid personality disorder are introverted reclusive, and clinically appear distant, aloof, apathetic, and emotionally detached [55], therefore they might not be easily prone to the emotional fluctuation including depression. Although Schizoid style did not contribute greatly to HCL-32 in BD I, it contributed in opposite direction to what it contributed in BD II. Whether the discrepancies were due to the HCL-32 heterogeneity remains unclear. Literature has not offered a direct support for the prediction of depression by the paranoid personality disorder functioning style, but the style was reported to be predicted by activity [56], and in clinics, the reduced activity is one of the typical depressive symptoms. The Borderline style predicted the MDQ scale, which fits into the results that patients with borderline personality disorder have elevated mood state [57].

The Passive-Aggressive and Schizoid (-) styles predicted the HCL-32 scale. Previous documentation has shown that the Passive-Aggressive personality disorder style was loaded together with Antisocial style [42], while the latter predicted HCL-32 in BD I, these results help to explain that the former positively predicted HCL-32 in BD II. Again there was no plausible explanation for the negative prediction of HCL-32 by the Schizoid personality disorder functioning style. The Schizoid trait was however, negatively related to the Openness to Experience and Conscientiousness traits [38], which were positively associated with mania symptoms [58]. These finding might be behind the negative association between Schizoid style and HCL-32 in BD II.

In healthy volunteers, together with Narcissistic (-) and Borderline styles predicted the PVP scale. Since the neuroticism trait is strongly related to depression [59, 60], and the former is often found higher in patients with borderline personality disorder [61]. Moreover, narcissistic personality disorder patients, who are preoccupied with fantasies of unlimited success, power, brilliance, beauty, or ideal love, have a grandiose sense of self-importance; they might demand excessive admiration, otherwise they might be depressed [62]. The Borderline and Dependent (-) styles predicted the MDQ scale. One of the borderline related traits, the impulsivity, is one of the manic features [63]. In addition, manic symptoms were predicted by the high conscientiousness trait [58], which had a negative relationship with the dependent personality disorder [38].

Our results of the different personality disorder functioning styles, emotional states, and their correlations in BD I and II also support a hypomanic personality and related cognitive styles in bipolar disorder reported previously [35–37]. The Borderline trait differentiates most between the groups, which is consistent with previous researches [33, 34]; the depression however, was influenced to a similar extent in BD I and II. Nevertheless, detailed personality styles and their relationship with the emotional states are different between BD I and II, which were not gender-driven either. Moreover, together with a previous investigation [20], we have demonstrated that the personality trait might only contribute to an increased likelihood of depression, but little to mania state, in BD I patients.

However, one should bear in mind two limitations of the present study. Firstly, the sample size of each bipolar disorder group is small which might be the reason that we failed to demonstrate a MDQ difference between BD II and the controls, or a PVP difference between BD I and the controls. We believe larger samples might help to clarify these concerns and to establish more stable relationships between personality disorders and mood states of these patients, and help to clarify whether there is a gender role played in these relationships. Secondly, we did not control the possible biological variations in our participants, therefore whether the personality styles and the emotional states have common denominators remains to be seen. Nevertheless, for the first time, we have confirmed the different predictabilities of the 11 functioning styles of personality disorder to the two types of bipolar disorder. In addition, we found that the prediction was more pronounced in BD II, which might be one reason that BD II has higher risk of suicide and poorer treatment outcome.
